# Phytochemical Analysis and Anticancer Activity of *Salvia chinensis Benth* in Colorectal Cancer: An Integrated Transcriptomic and Bioinformatic Study

**DOI:** 10.3390/ph19040569

**Published:** 2026-04-02

**Authors:** Long-Zhu Li, Xin-Yue Li, Zi-Yuan Wang, Tian-Qi Ma, Yan-Chao Wu, Hui-Jing Li

**Affiliations:** 1 Weihai Marine Organism & Medical Technology Research Institute, Harbin Institute of Technology, Weihai 264209, China; 23s030060@stu.hit.edu.cn (L.-Z.L.); 24b330009@stu.hit.edu.cn (X.-Y.L.); 23s030080@stu.hit.edu.cn (T.-Q.M.); ycwu@iccas.ac.cn (Y.-C.W.); 2 Department of Cell Biology, Harbin Medical University, 196 Baojian Road, Harbin 150081, China; 13234599256@163.com

**Keywords:** medicinal plants, *Salvia chinensis Benth*, anticancer, colorectal cancer, transcriptomics, bioinformatics, molecular docking

## Abstract

**Background/Objectives**: *Salvia chinensis Benth* (SJC), as a traditional medicinal plant, has garnered significant attention for its extensive pharmacological activities. However, a systematic investigation of its comprehensive chemical profile and the underlying mechanisms in colorectal cancer (CRC) remains to be elucidated. This study aims to elucidate the active compounds and targets responsible for the anti-colorectal cancer effects of the aqueous extract of SJC. **Methods:** An integrated strategy was employed. The chemical profile of the SJC aqueous extract was analyzed by UPLC-MS/MS. Its anticancer activities, including effects on cell proliferation, migration, and apoptosis, were evaluated using the HCT-116 CRC cell line. An integrated transcriptomic and bioinformatic approach, followed by protein–protein interaction (PPI) network analysis, was used to identify key molecular pathways and targets. Finally, molecular docking and cellular assays were performed to screen for potential bioactive compounds. **Results:** A total of 60 natural compounds were tentatively identified in SJC. SJC inhibited the proliferation, migration, and invasion of HCT-116 colorectal cancer cells. Through combined transcriptomic and bioinformatic analysis, four candidate key genes were initially identified. Further PPI network analysis prioritized CXCL8 as a key candidate among the four candidate targets. Molecular docking against CXCL8, together with subsequent cellular experiments, validated naringenin as a potential bioactive constituent contributing to the anti-CRC activity of SJC. **Conclusions:** This study provides a comprehensive chemical profile of SJC and offers significant insights into its potential anticancer mechanisms in CRC by identifying candidate targets and a potential bioactive constituent. While these findings are preliminary and require further experimental validation through additional CRC cell lines and in vivo models, they establish a solid foundation for future research into the therapeutic applications of SJC for colorectal cancer. These planned studies will help to further elucidate the underlying mechanisms and assess the translational potential of SJC.

## 1. Introduction

Colorectal cancer (CRC) is a major global health concern, ranking as the third most commonly diagnosed cancer and the second leading cause of cancer-related deaths worldwide [1]. While standard treatments like chemotherapy and targeted therapy have improved patient outcomes, significant clinical challenges remain, including acquired drug resistance and severe dose-limiting toxicities [2,3]. Although systemic therapy remains the standard of care for advanced CRC patients, the adverse effects associated with cytotoxic chemotherapy often result in suboptimal treatment outcomes. As a result, targeted therapies, which exhibit reduced toxicity toward healthy cells, are increasingly being utilized, particularly in cases of metastatic CRC [4]. Therefore, there is an urgent need to explore novel therapeutic agents with reduced toxicity and improved efficacy. Natural products, with their multi-target characteristics and favorable safety profiles, represent promising candidates for CRC treatment.

Research has shown that traditional Chinese medicine (TCM) plays a significant role in CRC management through multi-component, multi-target mechanisms [5,6]. For example, the ethanol extract of *Scutellaria baicalensis*, an herb belonging to the same family as SJC, inhibits CRC cell migration [7], while Jianpi Jiedu decoction exerts anti-CRC effects via the mTOR/HIF-1α/VEGF pathway [8]. Experimental studies indicate that TCM and its active components frequently exert anti-CRC effects by inducing apoptosis, modulating the cell cycle, and promoting autophagy [9].

*Salvia chinensis Benth* (Shijianchuan in Chinese, SJC) is a traditional medicine rich in bioactive constituents such as flavonoids, which underpin its established pharmacological properties [10,11]. In Traditional Chinese Medicine, *Salvia chinensis Benth* is most commonly consumed internally, typically as a decoction. While modern studies have confirmed SJC’s anticancer potential against various malignancies, including breast, liver, and pancreatic cancer [12], its specific application to colorectal cancer (CRC) remains underexplored. Although some active components have shown efficacy against gastrointestinal cancers, a systematic investigation is needed to identify the precise bioactive compounds within the whole SJC extract that target CRC and to delineate their potential molecular targets [11]. This knowledge gap limits its rational clinical application for CRC treatment.

Therefore, this study was designed to bridge this gap. Our central hypothesis is that SJC contains specific bioactive compounds that inhibit CRC cell proliferation by modulating critical oncogenic pathways. The primary objective was not to conduct an exhaustive mechanistic analysis, but rather to systematically identify these potent anti-CRC compounds and uncover their primary molecular targets. To achieve this, we employed an integrated strategy combining UPLC-MS/MS for chemical profiling, transcriptomics for unbiased target screening, and molecular docking coupled with in vitro assays to validate compound-target interactions.

## 2. Results

### 2.1. Identification of SJC Components

**Figure 1:**
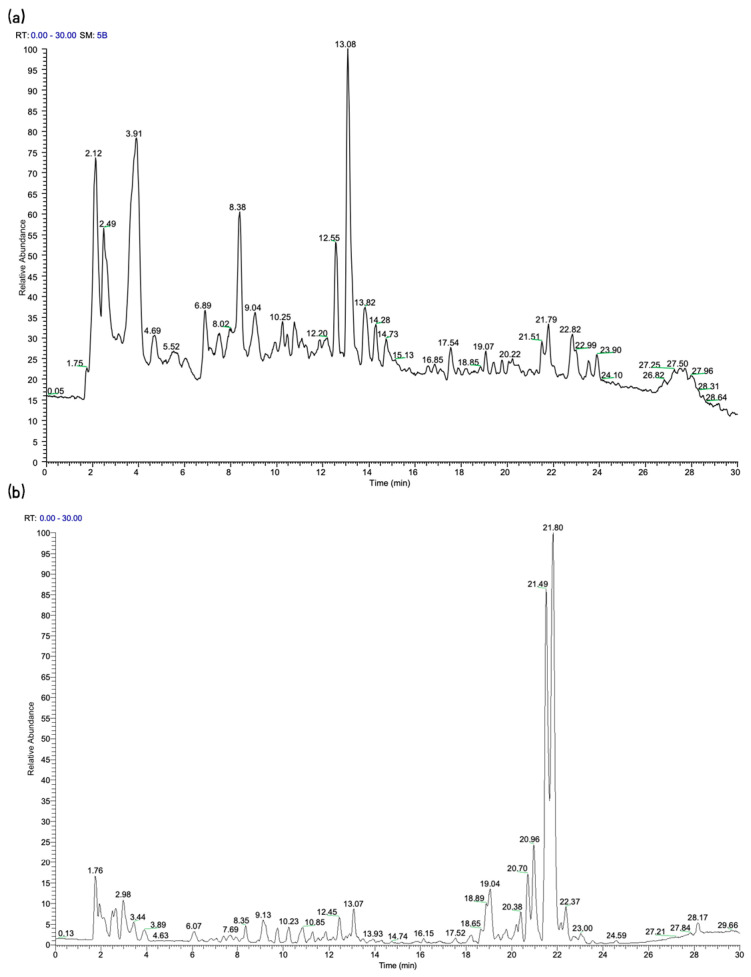
The total ion current (TIC) of SJC. (**a**) Positive ion mode. (**b**) Negative ion mode.

The characterization of chemical constituents in traditional Chinese medicine formulas is challenging due to their complex composition. High-throughput component analysis using UPLC-MS/MS provides an effective approach for the development of natural products. Figure 1a,b show the total ion current (TIC) chromatograms of SJC in positive and negative ion modes, respectively. Table S1 (Supplementary Material) summarizes the comprehensive information of the 60 compounds tentatively identified in SJC. To ensure the accuracy of content determination, a targeted MRM method was developed and validated for 9 representative compounds in SJC (Table 1). This selection was primarily determined by the commercial availability of their high-purity (>98%) certified reference standards, which is an essential requirement for establishing accurate calibration curves and ensuring the validity of the quantitative UPLC-MS/MS analysis.

### 2.2. Antitumor Activity of SJC

**Figure 2:**
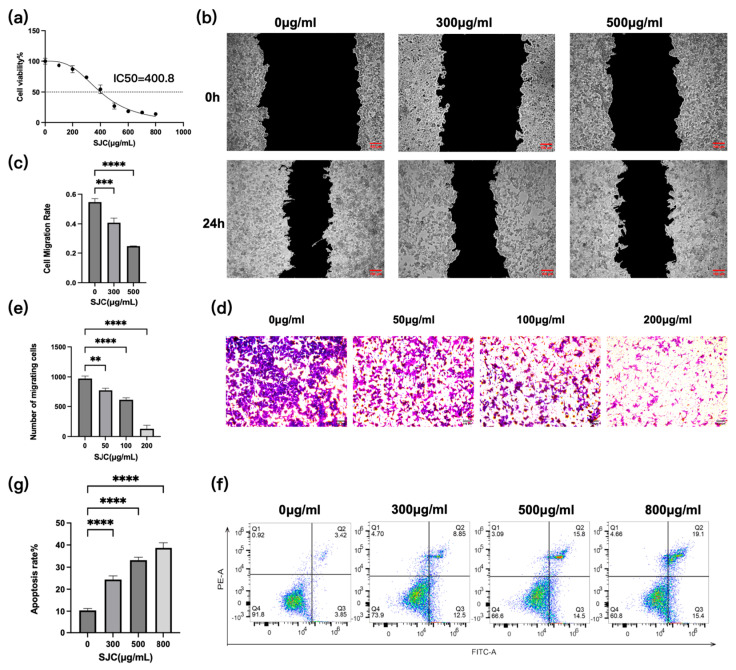
Verification of anticancer activity of SJC on HCT-116 colon cancer cells. (**a**) Curve of SJC inhibition of HCT-116 cell viability. (**b**) Scratch healing of HCT-116 cells. (**c**) Bar chart of SJC inhibition of HCT-116 cell scratch wound healing. (**d**) Transwell experiment cell migration. (**e**) SJC inhibition of HCT-116 cell longitudinal migration. (**f**) Effect of SJC on HCT-116 cell apoptosis. (**g**) SJC induction of HCT-116 cell apoptosis. ** for *p* < 0.01 and *** for *p* < 0.001 and **** for *p* < 0.0001 compared to the control group.

Figure 2a shows the viability of HCT-116 cells after 24 h treatment with various concentrations of SJC, with an IC_50_ value of 400.18 μg/mL, indicating that SJC exerts a certain inhibitory effect on the proliferation of HCT-116 cells. The wound-healing assay was used to evaluate lateral cell migration. The results showed that after 24 h of treatment, the scratch wound in the control group had healed (Figure 2b). Treatment with SJC at concentrations of 0, 300, and 500 μg/mL resulted in cell migration rates of 56%, 42%, and 24% (Figure 2c). The Transwell migration assay was employed to assess longitudinal cell migration. After 24 h of treatment with different SJC concentrations, methylene blue staining visualized the migration of HCT-116 cells (Figure 2d). Cell counting demonstrated that the average number of migrated cells was higher in the control group than in the SJC-treated groups (Figure 2e). Flow cytometry analysis further confirmed that the apoptotic rate of HCT-116 cells increased with rising SJC concentrations (Figure 2f). Treatment with SJC at concentrations of 0, 300, 500, and 800 μg/mL resulted in cell apoptosis rates of 9.4%, 26.1%, 33%, and 39.4%, respectively (Figure 2g). While these findings demonstrate the anti-CRC activity of SJC in HCT-116 cells, we acknowledge that the use of a single cell line limits the generalizability of the results. Future studies incorporating additional CRC cell lines will be necessary to assess potential cell line-specific variability and confirm the broader applicability of these observations.

### 2.3. HCT-116 Cell Transcriptomics Analysis

**Figure 3:**
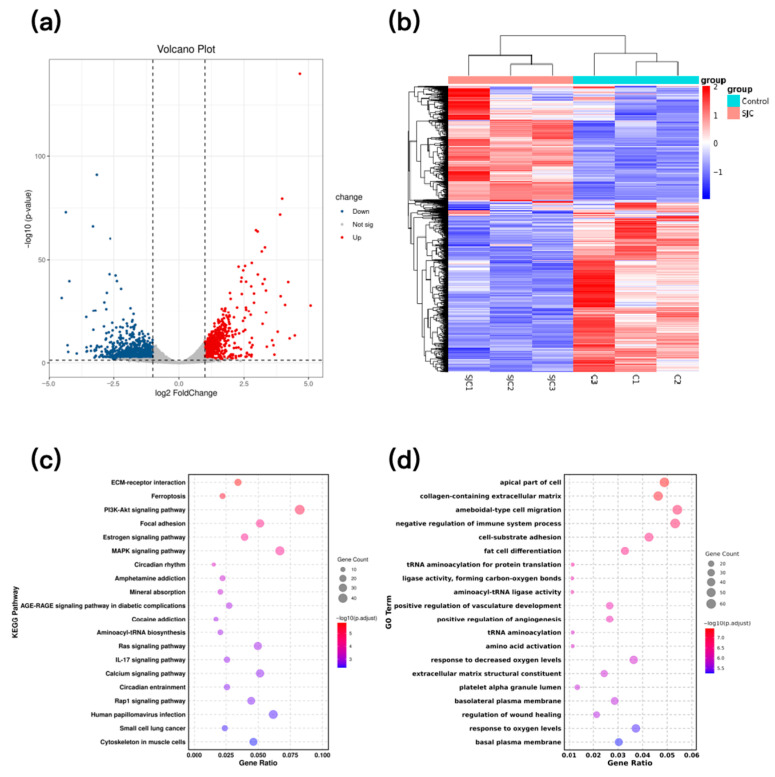
Transcriptome differential genes of HCT-116 cells. (**a**) Volcanic map of differential genes and differences. (**b**) Differential gene expression heatmap. (**c**) KEGG enrichment analysis. (**d**) GO enrichment analysis.

Transcriptomic analysis of SJC-treated HCT-116 cells identified 1787 differentially expressed genes, comprising 1183 upregulated and 604 downregulated genes (Figure 3a,b). Kyoto Encyclopedia of Genes and Genomes (KEGG) and Gene Ontology (GO) enrichment analyses of these differences revealed primary enrichment in signaling pathways such as ECM-receptor interaction, ferroptosis, PI3K-Akt, and MAPK (Figure 3c,d).

### 2.4. Candidate Target Screening

**Figure 4:**
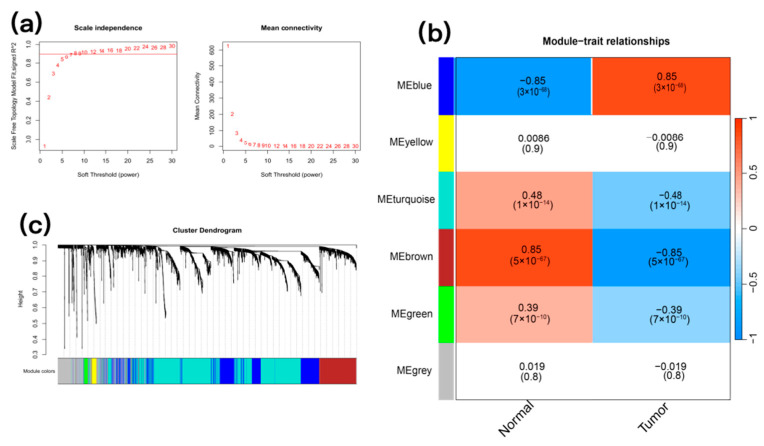
WGCNA analysis results of CRC. (**a**) Soft threshold selection. (**b**) CRC related gene clustering heatmap. (**c**) CRC related gene clustering tree diagram.

To prioritize drug-responsive genes with high clinical relevance, we implemented an integrative analysis strategy that connects our in vitro findings with patient-derived data. First, to identify gene networks central to CRC pathogenesis, we performed a Weighted Gene Co-expression Network Analysis (WGCNA) on a large, public CRC patient dataset. A soft-threshold power of 8 was selected to ensure a scale-free network (Figure 4a), resulting in the identification of several co-expression modules (Figure 4b,c). We focused on the blue and brown modules, as they were the most significantly associated with the clinical traits of CRC in the patient cohort. Next, to determine which of our drug-responsive genes are key players in these disease-relevant networks, we intersected the genes from the blue and brown modules with the list of differentially expressed genes (DEGs) from our SJC-treated cells. This intersection yielded 162 high-confidence overlapping genes, which represent promising candidates for mediating the anticancer effects of SJC and were thus carried forward for subsequent machine learning analysis.

**Figure 5:**
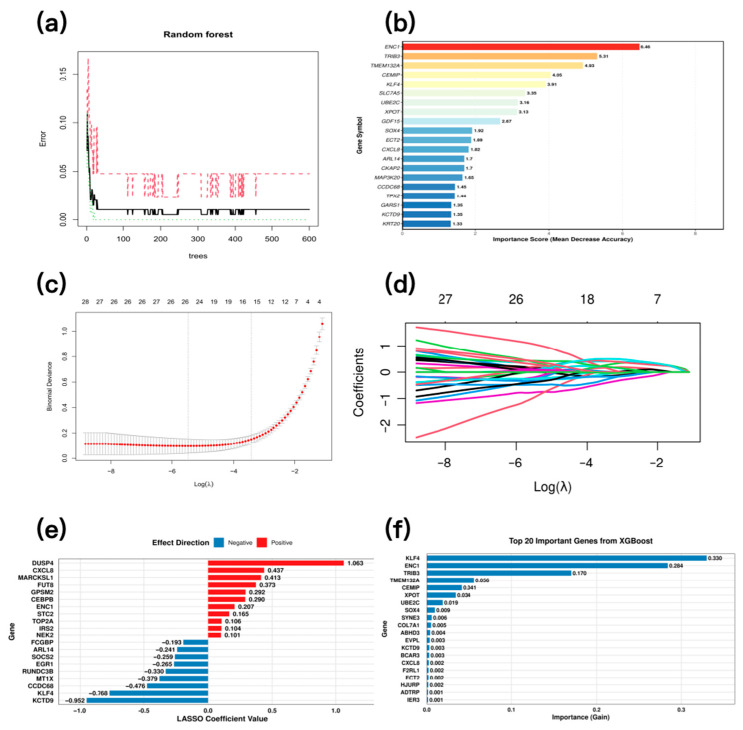
Candidate target selection using multiple machine learning algorithms. (**a**) Relationship between the number of trees in the random forest model and the classification error rate. (**b**) Feature gene importance ranking in the random forest model. (**c**) Misclassification error rate of the LASSO model. (**d**) Coefficient variation plot of feature variables in the LASSO regression model. (**e**) Top features selected by LASSO regression (**f**) Feature gene importance ranking in the XGBoost model.

To ensure rigorous model validation and address potential overfitting concerns, we further assessed the random forest model using stratified 10-fold cross-validation combined with recursive feature elimination (RF-RFE). A random forest model was initially constructed using 600 decision trees, achieving a stable out-of-bag (OOB) error rate of 0.42% (Figure 5a). To obtain a more reliable estimate of model performance, we applied RF-RFE within a 10-fold cross-validation framework. As shown in Supplementary Figure S1a, the cross-validation error reached its minimum when the top 20 most important genes were used. The final random forest model demonstrated strong discriminative ability, with an overall Area Under the ROC Curve (AUC) of 0.981 (Supplementary Figure S1b). The top 20 feature genes were selected based on importance scores (Figure 5b). Lasso regression identified the optimal penalty parameter (λ = 0.004) via least squares estimation (Figure 5c). This value minimized 10-fold cross-validation error and was selected as the optimal λ, identifying potential key targets (Figure 5d). Ultimately, 27 feature variables with non-zero coefficients were retained. Among the top 20 genes ranked by the absolute value of their regression coefficients, 11 were positively regulated and 9 were negatively regulated (Figure 5e). The XGBoost model achieved optimal performance at the 69th iteration. Receiver Operating Characteristic (ROC) analysis showed an AUC of 0.947 (>0.900) on the test set, indicating strong discriminative ability. The top 20 features were identified via Gini importance ranking (Figure 5f).

### 2.5. Candidate Targets Validation Results

**Figure 6:**
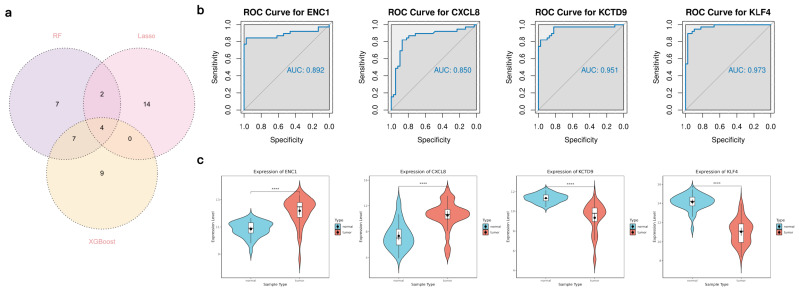
Candidate target validation in machine learning. (**a**) Intersection of multiple machine learning results yielded 4 candidate targets. (**b**) ROC curve analysis of candidate targets. (**c**) Violin plot of differential expression for candidate targets, **** for *p* < 0.0001 compared to the control group.

The final intersection of the three machine learning results identified four candidate targets for SJC treatment of CRC: ENC1, KLF4, CXCL8, and KCTD9 (Figure 6a). ROC analysis of these genes using the test set demonstrated excellent diagnostic performance, with AUC values of 0.892 for ENC1, 0.850 for CXCL8, 0.951 for KCTD9, and 0.973 for KLF4, exhibiting significant predictive potential for all four genes (Figure 6b). Violin plots illustrate the expression distributions of these genes between normal and tumor tissues, revealing that KLF4 and KCTD9 were significantly downregulated in tumor tissues (*p* < 0.0001), whereas ENC1 and CXCL8 were markedly upregulated (*p* < 0.0001) (Figure 6c). These results suggest all four genes may serve as candidate targets for CRC.

### 2.6. Screening of Potential Bioactive Compound

**Figure 7:**
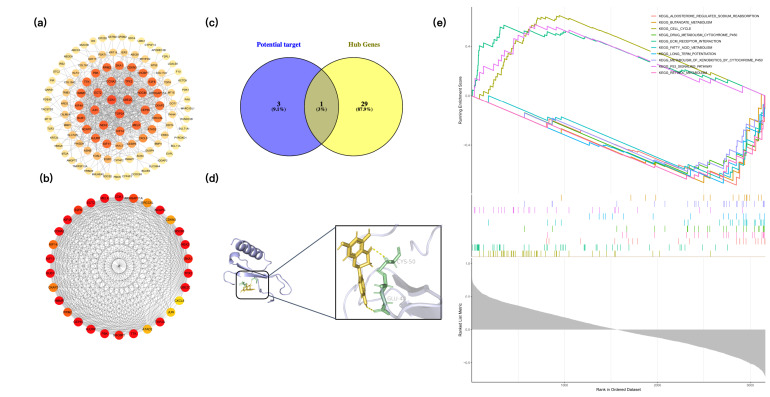
Identification of CXCL8 as a key candidate target and molecular docking validation. (**a**) PPI network of 117 key genes. (**b**) Top 30 hub genes. (**c**) Venn diagram showing the intersection of hub genes and four machine learning-derived targets. (**d**) Predicted binding mode of naringenin with CXCL8. (**e**) GSEA of CXCL8 in CRC.

A subset of 117 key genes was selected from the 162 common genes based on STRING interactions and the Maximal Clique Centrality (MCC) algorithm. These 117 genes were then used to construct a protein–protein interaction (PPI) network (Figure 7a). In this network, node size and color intensity are proportional to topological importance, as determined by the MCC score. From this network, the top 30 hub genes with the highest MCC scores were selected (Figure 7b). These hub genes were then intersected with the four potential target genes (KLF4, KCTD9, ENC1, and CXCL8) identified through machine learning, yielding a single overlapping gene: CXCL8 (Figure 7c).

Based on this target prioritization, molecular docking was subsequently performed between CXCL8 and the 60 active components identified in SJC. The binding affinities and interaction patterns were predicted, and calculated binding energies revealed that multiple compounds exhibited favorable interactions with CXCL8. Among the 60 compounds, naringenin exhibited the strongest docking affinity (−6.7 kcal/mol) (Figure 7d). To further validate the docking protocol and provide a reference for interpreting this result, we included ZINC21882765 as a positive control [13]. Under identical docking conditions, ZINC21882765 yielded a docking score of −7.59 kcal/mol, which is moderately more favorable than that of naringenin. This comparison indicates that the docking score of naringenin falls within a range comparable to a literature-reported CXCL8-binding candidate. The specific results of molecular docking can be found in Supplementary Material Table S2.

### 2.7. Gene Set Enrichment Analysis (GSEA)

To assess the impact of CXCL8 on CRC, samples were divided into high and low-expression groups based on the median expression levels, followed by GSEA. The significance thresholds were set at *p* < 0.05, FDR < 0.05, and |NES| > 1. GSEA revealed that multiple KEGG pathways were significantly enriched in association with high CXCL8 expression (Figure 7e). These included pathways associated with tumor progression, such as cell cycle, p53 signaling pathway, and ECM-receptor interaction, as well as metabolic pathways including fatty acid metabolism, retinol metabolism, butanoate metabolism, aldosterone-regulated sodium reabsorption, drug metabolism, and long-term potentiation. These findings suggest a broad impact of CXCL8 on both canonical oncogenic pathways and cellular metabolic homeostasis.

### 2.8. Validation of Naringenin Activity

**Figure 8:**
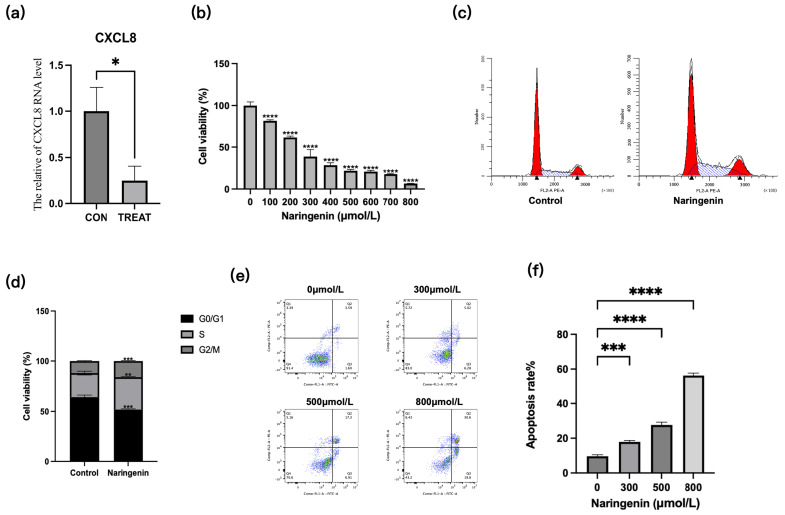
Experimental verification of naringenin as a potential bioactive compound in SJC. (**a**) RT-qPCR analysis of CXCL8 expression after naringenin treatment. (**b**) The effect of naringenin on the viability of HCT-116 cells. (**c**) The effect of naringenin on the cell cycle of HCT-116 cells. (**d**) Statistical analysis of the effect of naringenin on the cell cycle of HCT-116 cells. (**e**) The effect of naringenin on apoptosis of HCT-116 cells. (**f**) Statistical analysis of the effect of naringenin on apoptosis of HCT-116 cells. * for *p* < 0.05, ** for *p* < 0.01, *** for *p* < 0.001 and **** for *p* < 0.0001 compared to the control group.

To validate naringenin as a key anti-CRC inhibitor in SJC, we verified its effects on the expression of CXCL8 via RT-qPCR. Naringenin treatment induced downregulation of CXCL8 gene expression (*p* < 0.05) (Figure 8a). Functionally, cell viability assays demonstrated that naringenin could inhibit the proliferation of HCT-116 cells in a dose-dependent manner (Figure 8b). Cell cycle analysis showed that 300 μmol/L naringenin could increase the number of HCT-116 cells blocked in the S and G2/M phases from 37% to 49% (Figure 8c,d), which may be related to its regulation of cell cycle-related genes. Treatment with naringenin at concentrations of 0, 300, 500, and 800 μmol/L resulted in cell apoptosis rates of 8.6%, 17%, 27.4%, and 57.8%, respectively (Figure 8e,f).Therefore, naringenin’s anti-proliferative effect, mediated by target regulation and cell cycle arrest, is an important basis for its role as one of the identified potentially active compounds of SJC against CRC.

## 3. Discussion

SJC, a traditional Chinese herbal medicine, has long been recognized for its anticancer activity. In this study, UPLC-MS/MS was employed to characterize the chemical composition of SJC, revealing a rich content of flavonoids, phenolic acids, and alkaloids, which contribute to its potent therapeutic effects against various cancer cell lines. For example, flavonoids in SJC have been shown to promote apoptosis in hepatocellular carcinoma cells by inhibiting the NF-κB signaling pathway [10]. Additionally, epicatechol aldehyde in SJC influences the WT1 gene and may inhibit hepatocellular carcinoma by affecting the Wnt/β-catenin pathway [14]. Proteomic analyses have further demonstrated that SJC promotes autophagy in esophageal cancer cells via the AMPK/ULK1 signaling pathway, thereby suppressing their growth [11]. However, the molecular landscape affected by SJC in colorectal cancer (CRC) remains largely unexplored. To bridge this gap, our study first confirmed the anti-proliferative and anti-migratory effects of an SJC extract in HCT-116 CRC cells, the chemical profile of which was characterized by UPLC-MS/MS. These cellular assays demonstrated that SJC exerts moderate inhibitory effects on colorectal cancer cells. We then employed an integrated transcriptomic and bioinformatic approach not to definitively establish a mechanism, but to identify potential molecular targets and pathways that could explain its observed anticancer activity.

Through integrated transcriptomic and bioinformatic analyses, we initially identified four candidate targets, ENC1, KLF4, CXCL8, and KCTD9, as potential mediators of SJC’s anti-CRC effects. Among these, CXCL8 was prioritized as the key candidate target based on subsequent PPI network analysis. Consistent with previous studies, ENC1 has been implicated as a diagnostic marker in multiple tumors [15] and promotes CRC progression by upregulating β-catenin [16] and activating the JAK2-STAT5-AKT axis [17]. KLF4, another candidate, suppresses tumorigenesis by reducing β-catenin levels in thyroid cancer and colorectal cells [18,19]. CXCL8 (IL-8) is a well-known chemokine that facilitates neutrophil recruitment and cancer progression [20]; it is upregulated in various malignancies and has been proposed as a therapeutic target in prostate and thyroid cancers [21,22]. In CRC, CXCL8 promotes cell proliferation, migration, and invasion via the PI3K/Akt/NF-κB pathway [23], and its inhibition suppresses tumor growth and angiogenesis [24]. KCTD9, the fourth candidate, is negatively correlated with β-catenin in CRC tissues, suggesting a potential role in suppressing Wnt/β-catenin signaling [25]. The convergence of these genes in our initial screening highlights the multi-target nature of SJC, while the subsequent PPI-based prioritization of CXCL8 provides a focused entry point for mechanistic exploration.

To further explore the functional implications of CXCL8, we performed Gene Set Enrichment Analysis (GSEA) based on CXCL8 expression levels in CRC patients. High CXCL8 expression was significantly associated with enrichment of multiple KEGG pathways, including those linked to tumor progression, such as cell cycle, p53 signaling, and extracellular matrix (ECM)-receptor interaction, as well as metabolic pathways including fatty acid metabolism, retinol metabolism, butanoate metabolism, and drug metabolism (cytochrome P450 and xenobiotics metabolism) [26,27,28]. This pattern, where pathways involved in proliferation and metastasis are enriched alongside metabolic pathways, is consistent with the metabolic reprogramming characteristic of cancer cells [29]. The observed enrichment of drug metabolism pathways may have implications for chemotherapy response in CXCL8-high tumors [30]. Collectively, these results indicate that CXCL8 may contribute to CRC malignancy through multiple mechanisms, potentially involving both oncogenic signaling and metabolic remodeling. These pathway-level findings are consistent with our experimental observations that SJC and naringenin modulate cell cycle progression and induce apoptosis in HCT-116 cells, supporting the functional relevance of CXCL8-associated pathways.

Based on PPI network analysis, CXCL8 was prioritized as a key candidate among the four initially identified genes. The expression patterns of these four genes were validated using TCGA data, confirming their differential expression in CRC. Notably, CXCL8 showed a relatively stronger correlation with SJC-regulated transcriptomic changes and was therefore selected for further investigation. Molecular docking simulations were performed between the 60 compounds identified in SJC and CXCL8. Among these, naringenin exhibited a relatively favorable binding affinity to CXCL8. RT-qPCR experiments showed that naringenin treatment downregulated CXCL8 expression in HCT-116 cells. Together, these findings suggest that CXCL8 may represent a functional target of naringenin and SJC, warranting further investigation into the underlying molecular mechanisms.

Despite these findings, several limitations of the present study should be acknowledged. First, the compounds tentatively identified in the SJC aqueous extract were based solely on UPLC-MS/MS analysis without NMR confirmation and therefore remain tentative. Consequently, the biological activities observed with the whole extract cannot be definitively attributed to specific constituents, and potential synergistic interactions remain unexplored. Future isolation of individual bioactive compounds will be essential to investigate the synergistic effects and intrinsic relationships among the multiple components of SJC. Second, the molecular docking results are predictive only and do not constitute direct evidence of binding, while the proposed targets were identified through computational analyses and require further experimental validation through functional assays or direct binding studies. To further validate and extend these findings, future studies will incorporate additional colorectal cancer cell lines as well as in vivo models to assess the broader applicability of the observed effects. These limitations reflect common challenges in natural products research and do not diminish the core value of this study, which provides a comprehensive foundation for understanding the potential mechanisms of SJC against CRC and generates testable hypotheses for future investigations.

## 4. Materials and Methods

### 4.1. Materials

The whole plants of *Salvia chinensis Benth.* were collected from Guangdong Province, China in August 2024. A voucher specimen (No. SJC202408001) has been deposited at the Weihai Marine Organism & Medical Technology Research Institute, Harbin Institute of Technology, Weihai, China. Naringenin (4,5,7-trihydroxyflavanone) was purchased from Shanghai Aladdin Biochemical Technology Co., Ltd. (Shanghai, China; N107346, purity ≥ 98%). The study utilized an Ultimate3000 High-Performance Liquid Chromatography system and a Q-Exactive Focus Liquid Chromatography-Mass Spectrometry system (both from Thermo Fisher Scientific, Waltham, MA, USA). Absorbance was measured using a K3 microplate reader (Thermo Fisher Scientific, Waltham, MA, USA). Key reagents included methanol (Thermo Fisher Scientific, Waltham, MA, USA; anhydrous) and acetonitrile (Thermo Scientific, Shanghai, China; A955-4F), as well as dimethyl sulfoxide (DMSO) purchased from Sigma-Aldrich (Shanghai, China, 20–139).

### 4.2. Preparation of SJC Extract

The entire dried SJC plant was selected and crushed using a blender. To simulate the decoction method of SJC in traditional usage, SJC was accurately weighed to 20 g and transferred into a 500 mL beaker. Distilled water was added at a ratio of 1:20 (*w*/*v*), and the mixture was soaked in water at room temperature for 30 min and decocted at atmospheric pressure at 100 °C in a water bath for 1 h. It was then concentrated to 1/10 of its original volume using a rotary evaporator. From the concentrated extract, 5 mL was taken, mixed with 5 mL of anhydrous methanol, and stored at 4 °C overnight. The remaining concentrated extract was lyophilized in a freeze dryer, ultimately yielding 2.79 g of dry powder, corresponding to a yield of 13.8%. The final dried SJC extract was stored in a desiccator under sealed conditions.

### 4.3. Chromatographic and Mass Spectrometry Conditions

The methanol solution from Section 4.2, which had been stored overnight, was centrifuged at 5000 rpm for 15 min. The supernatant was collected and filtered through a 0.22 μm nylon membrane filter (Biosharp, Beijing, China; BS-QT-013).

Non-targeted metabolomic profiling was conducted using a Vanquish^TM^ UHPLC system coupled with a Q Exactive^TM^ Focus hybrid quadrupole-Orbitrap mass spectrometer (Thermo Fisher Scientific, Waltham, MA, USA). Chromatographic separation was achieved on a Waters ACQUITY UPLC BEH C18 column (Waters Corporation, Milford, MA, USA; 2.1 × 50 mm, 1.7 μm) maintained at 30 °C. The flow rate was set at 250 μL/min. The mobile phase consisted of water (A) containing 0.1% formic acid and acetonitrile (B). A linear gradient was applied as follows: 0–3 min, 3% B; 3–12 min, 3–40% B; 12–15 min, 40–65% B; 15–20 min, 65–100% B; 20–26 min, 100%; 26–28 min, 100–3% B; 28–30 min, 3% B.

The mass spectrometry conditions were set separately for each ionization mode. In positive ion mode, the ion spray voltage was set to 3.8 kV. In negative ion mode, it was set to 3.2 kV. The capillary temperature and auxiliary gas heater temperature were maintained at 320 °C in both modes. Data were acquired across a mass range of *m*/*z* 100–1500 Da in both positive and negative ion modes, yielding total ion current (TIC) chromatograms for each.

Data processing and compound identification were performed using Compound Discoverer software (version 3.2, Thermo Fisher Scientific). The raw data files (.raw) were processed for feature detection, peak alignment, and prediction of elemental compositions. A mass accuracy threshold of 5 ppm was applied for both molecular formula prediction and isotope pattern matching. For compound annotation, MS/MS spectra were matched against the mzCloud online spectral library. The following stringent criteria were applied for putative identification: a mass error of <5 ppm, a high degree of isotopic pattern matching, and an mzCloud best match score > 80. Finally, all potential identifications were manually curated by examining the precursor ion, retention time, and MS/MS fragmentation patterns to confirm assignments and remove duplicate entries.

To validate and quantify the chemical constituents identified in SJC extract, a SCIEX 6500+ triple quadrupole mass spectrometer (SCIEX, Framingham, MA, USA) was employed. Separation was performed on a Waters ACQUITY UPLC BEH C18 column (2.1 × 100 mm, 1.7 μm) at 40 °C. The mobile phase consisted of 0.1% formic acid in water (A) and acetonitrile (B) with a specific 30 min gradient program: 0–24.0 min, 5–95% B; 24.0–24.9 min, 95% B; 24.9–25.0 min, 95–5% B; and 25.0–30.0 min, 5% B. The flow rate was 0.3 mL/min, and the injection volume was set at 2 μL. Detection was executed in Multiple Reaction Monitoring (MRM) mode, scanning in both positive and negative ionization modes to ensure comprehensive metabolite coverage. Electrospray ionization (ESI) source parameters were optimized as follows: curtain gas, 10 psi; collision gas, 8 psi; ion source gas 1, 16 psi; ion source gas 2, 20 psi; source temperature, 500 °C; and ion spray voltage, ±4500 V. Data were acquired and processed using Analyst and MultiQuant MD 3.0.3 software.

### 4.4. CRC Microarray Data Processing

In this study, the GSE41258 dataset was obtained from the Gene Expression Omnibus (GEO, https://www.ncbi.nlm.nih.gov/geo/ (accessed on 21 December 2024)), with microarray data derived from the GPL96 platform (Affymetrix, Inc., Santa Clara, CA, USA; Human Genome U133A Array). Following the annotation file requirements, probes were converted to gene symbols, and low-expression samples were excluded. Ultimately, 54 normal colon samples and 186 primary tumor samples from GSE41258 were selected, resulting in a total of 240 samples. This dataset was used as the training set for subsequent co-expression analysis and machine learning model development.

RNA sequencing data for the CRC cohort were obtained from the Cancer Genome Atlas (TCGA). Raw count data were processed and normalized to transcripts per million (TPM) values. After filtering to retain protein-coding genes and excluding low-quality samples, 39 tumor samples and their 39 matched adjacent normal tissues were selected as the independent validation set. This paired design was chosen to minimize false positives that could arise from sample heterogeneity, thereby enhancing the reliability of the validation.

### 4.5. Cell Culture

HCT-116 cells (a human colon cancer cell line) were obtained from the Stem Cell Bank, Chinese Academy of Sciences (Serial: SCSP-5076). The cells were cultured in high-glucose DMEM supplemented with 10% fetal bovine serum (FBS) and 1% penicillin-streptomycin. The high-glucose DMEM medium was purchased from Solarbio (Beijing, China).

### 4.6. Cell Viability Assay

Cell viability was assessed using the MTT assay. MTT is a pale yellow, water-soluble tetrazolium salt that can be reduced by succinate dehydrogenase in the mitochondria of living cells to form insoluble purple formazan crystals. HCT-116 cells were seeded in a 96-well plate at a density of 5 × 10^3^ cells/well, with 150 μL of culture medium per well. The blank control group was cultured in high-glucose DMEM, while the experimental groups were treated with SJC at concentrations of 100, 200, 300, 400, 500, 600, 700, and 800 μg/mL. After 24 h of incubation, 15 μL of MTT solution (5 mg/mL) was added to each well. The plates were then incubated at 37 °C for 4 h. Subsequently, the supernatant was carefully removed, and 150 μL of DMSO was added to each well to dissolve the formazan crystals. The absorbance of each well was measured at 560 nm, and cell viability was calculated using the following formula:

$$C=\frac{A_{2}}{A_{1}}\times 100\%$$

*C*—Cell viability (%).
*A*_1_—Absorbance of the control group.
*A*_2_—Absorbance of the experimental group.

### 4.7. Wound-Healing Assay

Cells were seeded at an appropriate density in 6-well plates for culture. After 24 h, the cells were treated with different concentrations of SJC and cultured until a confluent monolayer had formed. A uniform scratch was then created in the monolayer using a sterile pipette tip. The scratch area was gently rinsed twice with PBS to remove detached debris. Thereafter, the monolayer was maintained in serum-free medium. Wound closure was observed at the same location at 0 h and 24 h using an inverted microscope, and changes in the scratch area were quantified and analyzed using ImageJ software (Version 1.53k).

### 4.8. Transwell Assay

Cell migration assays were conducted using Transwell inserts (8 μm pore size; NEST, Wuxi, Jiangsu, China). A total of 1 × 10^5^ HCT-116 cells were suspended and seeded into the upper chamber. The control group was treated with 300 μL of serum-free DMEM medium, while the experimental groups were treated with 300 μL of SJC at concentrations of 50, 100, and 200 μg/mL. The lower chamber was filled with medium supplemented with 8% fetal bovine serum. After 24 h of incubation, cells adhering to the bottom and sides of the upper chamber were fixed with methanol for 20 min and stained with 0.1% crystal violet for 25 min. Migrating cells were observed and photographed under a fluorescence microscope, and the number of cells was quantified using ImageJ software for statistical analysis.

### 4.9. Apoptosis Assay

Cell apoptosis was assessed using an Annexin V-FITC/PI apoptosis detection kit (Beyotime, Shanghai, China; C1062M) according to the manufacturer’s instructions. Briefly, cells were subjected to various treatments upon reaching 80–90% confluence in six-well plates. Subsequently, the cells were harvested, washed twice with PBS, and resuspended in 400 μL of binding buffer. Annexin V-FITC/PI was added to the cell suspension, which was then incubated at 37 °C in the dark for 30 min. Apoptotic cells were quantified using a BD Accuri C6 Plus flow cytometer (BD Biosciences, San Jose, CA, USA).

### 4.10. WGCNA Analysis in CRC Patients

WGCNA is an algorithm designed to identify gene modules that are highly correlated with specific phenotypes or traits. In this study, the WGCNA R package (Version 1.72) was used to construct a gene co-expression network and identify hub genes within co-expressed gene modules using the GSE41258 dataset. Outlier samples were excluded, and the top 25% of genes with the highest variance were selected for further analysis. An appropriate soft threshold power (β) was then determined. Using the optimal soft threshold, a weighted network was constructed by converting the adjacency matrix into a topological overlap matrix, and modules were identified using the dynamic pruning algorithm. Finally, gene significance (GS) and module significance (MS) were calculated to evaluate the relevance of genes to biological modules and their association with clinical information. Modules exhibiting the strongest intergroup differences were selected as potential genes involved in CRC pathogenesis. These genes, representing clinically relevant CRC-associated genes, were retained for subsequent integration with transcriptomic data from SJC-treated cells.

### 4.11. Transcriptomic Analysis of HCT-116

HCT-116 cells were cultured overnight in cell culture dishes at a density of 3 × 10^5^ cells/well. The experiment included two groups: a control group and an SJC-treated group, each with three biological replicates. The control group was cultured in complete DMEM medium, while the SJC-treated group received complete DMEM medium supplemented with 500 μg/mL SJC. Total RNA was extracted using TRIzol reagent. RNA preparation and sequencing were performed by Novogene (Beijing, China). Specifically, total RNA was enriched using Oligo dT beads (Novogene, Beijing, China), and cDNA libraries were constructed. The quality of the cDNA libraries was assessed using an Agilent 5400 Bioanalyzer (Agilent Technologies, Santa Clara, CA, USA). After passing quality control, the libraries were sequenced on a NovaSeq 6000 platform (Illumina, San Diego, CA, USA). To ensure the robustness and reliability of the data, a series of quality-control and filtering steps were implemented. Initially, low-quality sequencing reads were removed from the raw data to obtain high-quality clean reads. For the differential expression analysis, a pre-filtering step was applied where genes with expression levels below 1 were excluded to reduce noise. The analysis was then performed using the DESeq2 R package (Version 1.38.3). Differentially expressed genes (DEGs) were identified under stringent criteria of an absolute |log2FC| ≥ 1 and an adjusted *p*-value < 0.05, a method that effectively controls the false discovery rate. Visualization of DEGs was subsequently carried out using the ggpubr (Version 0.6.0)and pheatmap R packages (Version 1.0.12), and KEGG and GO enrichment analyses of the identified DEGs were conducted using the ClusterProfiler R package (Version 4.6.2). These DEGs, representing genes responsive to SJC treatment, were then intersected with the clinically relevant genes obtained from WGCNA to prioritize candidate targets for further analysis.

### 4.12. Machine Learning-Based Screening of Candidate Genes in CRC

Based on the intersection of clinically relevant genes identified by WGCNA and SJC-regulated DEGs from RNA-seq analysis, a candidate gene set was obtained. This set represents genes that are both associated with CRC pathogenesis and responsive to SJC treatment and was used as input features for machine learning modeling. To ensure the rigor and reproducibility of the feature selection process and to mitigate the risk of overfitting, we implemented specific validation and hyperparameter tuning strategies for each algorithm. The LASSO regression model was implemented using the glmnet R package (Version 4.1-8), where the optimal regularization parameter (lambda) was determined via 10-fold cross-validation. We selected the lambda.min value, which corresponds to the minimum mean cross-validated error, to explicitly control for overfitting, and genes with non-zero coefficients were identified as important features. For the Random Forest model, constructed with the randomForest package, we set the number of trees (ntree) to 600. To obtain a robust estimation of feature importance, we implemented a 10-fold cross-validation framework where the importance scores, ranked by the Mean Decrease in Gini index, were averaged across all 10 folds. The XGBoost model was trained using the xgboost package with a maximum tree depth of 6. To control for overfitting, an early stopping strategy was adopted based on the Root Mean Square Error (RMSE); training was halted if the RMSE, monitored on a separate validation dataset, failed to decrease for 20 consecutive rounds, and feature importance was derived from the Gain metric. Finally, to identify the most robust gene targets, we took the intersection of the top 20 genes selected by each of the three models.

### 4.13. External Dataset Validation of Candidate Genes

The candidate targets identified in Section 4.12 were validated using an external dataset. From the COAD dataset within the TCGA database, 39 normal samples along with 39 matched COAD samples were selected to form the validation set. Differential expression of the candidate targets was assessed using ROC curves and violin plots.

### 4.14. PPI Network Construction

A protein–protein interaction (PPI) network was constructed using the STRING database. The network was analyzed using Cytoscape software (version 3.9.1) to identify central hub genes via the Maximal Clique Centrality (MCC) algorithm.

### 4.15. Molecular Docking

Key protein structures were retrieved from the RCSB PDB database (https://www.rcsb.org/ (accessed on 7 September 2025)). AutoDock Tool 1.5.6 was used to remove small molecules and water molecules, hydrogen atoms were added, and charges were calculated. Structures of active ingredients were obtained from the PubChem database. AutoDock Tool was used to balance the charges of the active ingredients, while molecular docking was performed using AutoDock Vienna. The conformation with the lowest binding energy (indicating the highest affinity) was selected, exported, and visualized using PyMol 3.1.4.1.

### 4.16. GSEA

GSEA was performed to identify biological pathways associated with the candidate targets. Initially, the correlation between the candidate targets and all other genes in the WGCNA network was calculated. All genes were then ranked based on their correlation coefficients in descending order. The KEGG subset was used as the reference gene set for enrichment analysis, with a *p*-value < 0.05 considered statistically significant.

### 4.17. Cell Cycle Assays

Cell cycle was evaluated using a cell cycle detection kit (C1052, China, Beyotime) according to the manufacturer’s instructions. At 48 h of treatment, cells were incubated with RNaseA and propidium iodide in the dark for 30 min.

### 4.18. RT-qPCR

Total RNA was isolated from both control and treated samples using TRIzol reagent. Subsequently, 300–500 ng/μL of total RNA was reverse transcribed into cDNA using the PrimeScript^TM^ RT Kit (Takara, Shiga, Japan). Primers were designed with NCBI to ensure amplification of single products without non-specific peaks. These primers were used for further amplification. mRNA levels were quantified by SYBR Premix Ex Taq (Takara, Shiga, Japan). Detailed primer sequences are shown in Table 2.

### 4.19. Statistical Analysis

All statistical analyses were performed using GraphPad Prism (version 10.0) and R software (version 4.2.1). Each experiment was performed with at least three independent biological replicates unless otherwise specified. Statistical comparisons between two groups were performed using an unpaired Student’s *t*-test, while comparisons among multiple groups were conducted using a one-way Analysis of Variance (ANOVA) followed by Tukey’s post hoc test. Detailed information regarding the specific statistical methods used for each experiment, including sample sizes and exact *p*-values where applicable, is provided in the corresponding figure legends and results sections.

## 5. Conclusions

In this study, we demonstrate that the aqueous extract of SJC exhibits potential anticancer effects against CRC by inducing apoptosis and inhibiting cell migration and proliferation. Through integrated transcriptomic and bioinformatic analyses, four candidate targets, ENC1, KLF4, CXCL8, and KCTD9, were identified as potential mediators of these effects, with CXCL8 emerging as a prioritized target based on subsequent PPI network analysis. Molecular docking simulations and RT-qPCR validation further suggested that naringenin, a compound identified in the SJC aqueous extract, may contribute to the observed anti-CRC activity through modulation of these targets. In summary, this study provides a comprehensive chemical profile of the SJC aqueous extract and offers preliminary insights into its potential multi-target mechanisms against CRC. These findings establish a foundation for future investigations, including validation in additional CRC cell lines and in vivo models, to further explore the therapeutic potential of SJC.

## Figures and Tables

**Table 1:** Quantification and method validation parameters for 9 bioactive compounds in SJC.

Analyte	Equation	R^2^	SJC (μg/g)
Caffeic acid	y = 4550.3x − 10,651	0.9997	6.309 ± 0.653
Vanillin	y = 44,267x − 96,667	0.9991	0.196 ± 0.008
Quercetin	y = 4038.9x − 11,187	0.9994	0.244 ± 0.075
Naringenin	y = 144,574x + 138,380	0.9983	0.110 ± 0.004
Chlorogenic acid	y = 182,003x + 367,152	0.9972	1.058 ± 0.069
Rutin	y = 117,281x + 238,193	0.9992	5.903 ± 0.152
Salicylic acid	y = 67,775x + 319,891	0.9980	0.332 ± 0.011
Suberic acid	y = 13,423x + 106,236	0.9960	0.035 ± 0.003
Citric acid	y = 4005.8x − 17,534	0.9976	15.723 ± 0.540

**Table 2:** Primer sequences for RT-qPCR.

Gene Name	Forward Primer	Reverse Primer
CXCL8	GGAGAAGTTTTTGAAGAGGGCTG	ACAGACCCACACAATACATGAAG

## Data Availability

The original contributions presented in this study are included in the article and Supplementary Material. Further inquiries can be directed to the corresponding author.

## Supplementary Materials

The following supporting information can be downloaded at: https://www.mdpi.com/article/10.3390/ph19040569/s1, Table S1: Compound information identified in SJC; Table S2: SJC active ingredient molecular docking results. Figure S1: Rigorous validation of the random forest model.

## Abbreviations

The following abbreviations are used in this manuscript:

SJC	*Salvia chinensis Benth*
CRC	Colorectal cancer
TCM	Traditional Chinese medicine
TIC	Total ion current
KEGG	Kyoto Encyclopedia of Genes and Genomes
GO	Gene Ontology
WGCNA	Weighted Gene Correlation Network Analysis
GSEA	Gene Set Enrichment Analysis
GEO	Gene Expression Omnibus
TCGA	The Cancer Genome Atlas
TPM	Transcripts per million
DEGs	Differentially expressed genes
