# Supplementary material for: Phytochemical Analysis and Anticancer Activity of Salvia chinensis Benth in Colorectal Cancer: An Integrated Transcriptomic and Bioinformatic Study
Source: Pharmaceuticals (Basel). 2026 Apr 2;19(4):569. doi: 10.3390/ph19040569 (PMC13118775; doi:10.3390/ph19040569)
Supplement: Supplementary file 1 [file pharmaceuticals-19-00569-s001.zip › pharmaceuticals-4182419-supplementary.pdf]

Supplementary Material

Table S1. Compound information identified in SJC.
Table S2. SJC active ingredient molecular docking results.
Figure S1. Rigorous validation of the random forest model.

Table S1. Compound information identified in SJC.

Name	Formula	FragmentIons	Annot.DeltaMass[ppm]	m/z	RT[min]	ReferenceIon
Caffeic acid	$C_9H_8O_4$	136.04443，117.03378，91.05429，89.03880	-1.06	179.03476	8.391	[M-H]-1
Hexadecanamide	$C_{16}H_{33}NO$	116.10743，102.09166，88.07635，57.07064	0.71	256.26367	28.047	[M+H]+1
Apigenin 7-O-glucuronide	$C_{21}H_{18}O_{11}$	271.06730，225.05539，153.01842，119.04948	1.45	447.09283	12.65	[M+H]+1
DEET	$C_{12}H_{17}NO$	109.06625，100.07620，91.05478，72.08150	1.57	192.13859	18.666	[M+H]+1
Xylitol	$C_5H_{12}O_5$	149.04488，119.03425，101.02356，89.02350，71.01287	-0.23	151.06094	1.968	[M-H]-1
Ferulic acid	$C_{10}H_{10}O_4$	178.02667，149.06013，134.03659，106.04160	-1.47	193.05031	8.319	[M-H]-1
6-Methylquinoline	$C_{10}H_9N$	143.07327，128.04968，117.05754，115.05466，91.05485	3.02	144.08121	8.195	[M+H]+1
Syringic acid	$C_9H_{10}O_5$	181.04984，156.07046，140.04691，95.04970	1.72	199.06044	9.794	[M+H]+1
Glutaric acid	$C_5H_8O_4$	113.02364，87.04422，69.03360	-4.23	131.03442	3.097	[M-H]-1
Chlorogenic acid	$C_{16}H_{18}O_9$	191.05585，161.02391，133.02902，87.00787	1.96	353.08853	5.425	[M-H]-1
Pimelic acid	$C_7H_{12}O_4$	141.05493，115.07566，97.06499	-2.56	159.06587	8.57	[M-H]-1
2,4,6-Trihydroxyacetophenone	$C_8H_8O_4$	149.02373，123.04440，121.02863，108.02100	-2.46	167.03455	3.918	[M-H]-1
Gluconic acid	$C_6H_{12}O_7$	141.01875，129.01862，75.00780，59.01283	1.55	195.05133	1.878	[M-H]-1
Kynurenic acid	$C_{10}H_7NO_3$	162.05510，144.04535，116.04983	1.74	190.05028	6.819	[M+H]+1
Quercetin	$C_{15}H_{10}O_7$	257.04465，201.05472，153.01843，137.02353	1.41	303.05035	11.268	[M+H]+1
3-Phenyllacticacid	$C_9H_{10}O_3$	147.04449,119.04944,103.05441,72.99212	-277039.24	165.05528	11.09	[M+FA-H]-1
DL-Mali acid	$C_4H_6O_5$	115.00283,89.02348,72.99210,71.02183	-5.02	133.01358	2.248	[M-H]-1
2,3,4,9-Tetrahydro-1H-β-carboline-3-carboxylic acid	$C_{12}H_{12}N_2O_2$	171.09225，144.08093，130.06549，117.07011	2.71	217.09775	8.196	[M+H]+1
Oleamide	$C_{18}H_{35}NO$	265.25296,247.24232,135.11710,109.10159	-60532.93	282.27948	27.999	[M+NH4]+1

Asiatic acid	$C_{30}H_{48}O_5$	435.33264,425.34753,247.16946,163.1119	-36878.48	471.34784	16.624	[M+H]+1
4-Pyridoxic acid	$C_8H_9NO_4$	166.05013，138.05521，120.04473，92.05022	1.77	184.06096	2.623	[M+H]+1
Valylproline	$C_{10}H_{18}N_2O_3$	116.07102，72.08150，70.06586，55.05502	2.8	215.13962	2.844	[M+H]+1
Methyl cinnamate	$C_{10}H_{10}O_2$	131.04941，107.08588，95.04976	1.09	163.0755	10.422	[M+H]+1
Indole	C_8H_7N	118.06549,117.05784,91.05485	3.96	118.06559	12.746	[M+H]+1
Corymboside	$C_{26}H_{28}O_{14}$	511.12363，427.10355，379.08170，295.06049	1.91	565.15607	9.899	[M+H]+1
Methylsuccinic acid	$C_5H_8O_4$	113.02361,87.04422,69.03356	-4.34	131.03441	4.15	[M-H]-1
Berberine	$C_{20}H_{17}NO_4$	321.09982	1.58	336.12357	14.398	[M+H]+1
Suberic acid	$C_8H_{14}O_4$	120.09138，111.08069，83.04927，80.02638	-2.15	173.08156	11.009	[M-H]-1
3-Hydroxy-3-(methoxycarbonyl)pentanedioic acid	$C_7H_{10}O_7$	143.03438，111.00795，83.00783，72.99213	0.16	205.03545	2.978	[M-H]-1
Naringenin	$C_{15}\ H_{12}\ O_5$	171.02890，153.01823，147.04406，119.04914	1.55	272.0689	12.451	[M+H]+1
3-(2-Hydroxyethyl)indole	$C_{10}H_{11}NO$	144.08096,130.06558,11.07027	0.56	162.09143	13.707	[M+H]+1
Methyl isonicotinate	$C_7H_7NO_2$	110.06053,108.04494,80.05012,67.04244	-124235.24	138.05534	4.788	[M+NH4]+1
Andrographolide	$C_{20}H_{30}O_5$	295.18570,213.12825,145.10179,99.04494,81.07045	-2.14	351.21585	16.936	[M+H]+1
4-Acetamidobutanoic acid	$C_6H_{11}NO_3$	128.07088,104.07108,87.04468,86.06068	2.43	146.0815	2.679	[M+H]+1
Terephthalic acid	$C_8H_6O_4$	121.02873,93.03362	-2.48	165.01892	8.333	[M-H]-1
2-Butoxyacetic acid	$C_6H_{12}O_3$	113.02354,85.06494,75.00768,72.99216	-4.52	131.07077	9.505	[M-H]-1
Leucylproline	$C_{11}H_{20}N_2O_3$	116.07101,86.09706,70.06585	2.31	229.1552	5.532	[M+H]+1
Vanillic acid	$C_8H_8O_4$	152.01091,123.00809,108.02082,95.01299	-2.5	167.03456	10.899	[M-H]-1
(+/-)12(13)-DiHOME	$C_{18}H_{34}O_4$	295.22784,277.21686,213.14827,183.13866	2.61	313.23904	21.445	[M-H]-1
Dibutyl phthalate	$C_{16}H_{22}O_4$	205.08611,167.03415,149.02354,121.02869	1.91	279.15961	25.231	[M+H]+1

Citric acid	$C_6H_8O_7$	173.00911,129.01862,101.02361	-1.09	191.01952	8.404	[M-H]-1
Phenethylamine	$C_8H_{11}N$	105.07039,103.05482	-140607.15	122.09693	5.109	[M+NH4]+1
Apigenin	$C_{15}H_{10}O_5$	243.06622,225.05499,163.03891,119.04945	0.7	271.06021	16.599	[M+H]+1
Salicylic acid	$C_7H_6O_3$	93.03368,65.03868	-4.89	137.02374	12.57	[M-H]-1
DL-Tryptophan	$C_{11}H_{12}N_2O_2$	188.07082,170.06010,146.06020,118.06549	2.31	205.09763	6.096	[M+H]+1
Naringin	$C_{27}H_{32}O_{14}$	459.11600,271.06149,151.00308,125.02377	2.28	579.17316	12.452	[M-H]-1
Succinic acid	$C_4H_6O_4$	73.02853,55.01787	-5.81	117.01865	2.595	[M-H]-1
Rutin	$C_{27}H_{30}O_{16}$	303.05002,229.04979,153.01851,129.05482	1.07	611.16101	10.852	[M+H]+1
(+/-)9(10)-EpOME	$C_{18}H_{32}O_3$	277.21765,171.10129	1.37	295.22827	23.881	[M-H]-1
3-[(1-Carboxyvinyl) oxy]benzoic acid	$C_{10}H_8O_5$	179.03462,163.03937,137.02370	-0.67	207.02975	12.572	[M-H]-1
Azelaic acid	$C_9H_{16}O_4$	169.08670,143.10707,123.08086	-1.64	187.09727	13.26	[M-H]-1
Nicotinamide	$C_6H_6N_2O$	106.02930,95.04508,80.05019	3.63	123.05573	2.464	[M+H]+1
Cryptotanshinone	$C_{19}H_{20}O_3$	279.13843,251.14339,237.09209	2.12	297.14911	23.924	[M+H]+1
Gentisic acid	$C_7H_6O_4$	109.02867,81.03367	-3.07	153.01886	4.696	[M-H]-1
Indole-3-acrylic acid	$C_{11}H_9NO_2$	170.06026,146.06020,118.06549	2.27	188.07103	6.1	[M+H]+1
DL-Tartaric acid	$C_4H_6O_6$	130.99754,105.01851,103.00286,75.00772	-2.99	149.00871	2.181	[M-H]-1
3-Hydroxypicolinic acid	$C_6H_5NO_3$	122.02444,112.03970,94.02928	1.8	140.03447	2.47	[M+H]+1
Catechol	$C_6H_6O_2$	108.02085,91.01790	-6.52	109.02879	4.687	[M-H]-1
Adenosine	$C_{10}H_{13}N_5O_4$	136.06197,119.03539,94.04093,69.03422	1.5	268.10443	2.47	[M+H]+1
Isoleucine	$C_6H_{13}NO_2$	86.09708,69.07070	3.32	132.10234	2.664	[M+H]+1

Table S2. SJC active ingredient molecular docking results (kcal/mol).

	**CXCL8**
Caffeic acid	-4.5
Hexadecanamide	-5.78
Apigenin 7-O-glucuronide	-6.02
DEET	-4.86
Xylitol	-4.4
Ferulic acid	-5.03
6-Methylquinoline	-4.49
Syringic acid	-4.57
Glutaric acid	-4.41
Chlorogenic acid	-5.16
Pimelic acid	-4.73
2,4,6-Trihydroxyacetophenone	-4.2
Gluconic acid	-4.56
Kynurenic acid	-4.72
Quercetin	-5.07
3-Phenyllactic acid	-4.8
DL-Malic acid	-4.01
2,3,4,9-Tetrahydro-1H-β-carboline-3-carboxylic acid	-4.61
Oleamide	-5.61
Asiatic acid	-5.29
4-Pyridoxic acid	-4.32
Valylproline	-4.92
Methyl cinnamate	-4.42
Indole	-4.1
Corymboside	-6.49
Methylsuccinic acid	-4.36
Berberine	-5.99
Suberic acid	-4.8
3-Hydroxy-3-(methoxycarbonyl)pentanedioic acid	-4.58
Naringenin	-6.7
3-(2-Hydroxyethyl) indole	-4.41
Methyl isonicotinate	-4
Andrographolide	-5.17
4-Acetamidobutanoic acid	-4.47
Terephthalic acid	-4.28
2-Butoxyacetic acid	-4.59
Leucylproline	-4.83

Vanillic acid	-4.46
(+/-)12(13)-DiHOME	-5.96
Dibutyl phthalate	-5.62
Citric acid	-4.46
Phenethylamine	-3.98
Apigenin	-5.27
Salicylic acid	-4.31
DL-Tryptophan	-5.1
Naringin	-5.17
Succinic acid	-4.18
Rutin	-6.66
(+/-)9(10)-EpOME	-5.86
3-[(1-Carboxyvinyl) oxy]benzoic acid	-5.05
Azelaic acid	-5.27
Nicotinamide	-3.88
Cryptotanshinone	-4.97
Gentisic acid	-4.22
Indole-3-acrylic acid	
DL-Tartaric acid	-4.74
3-Hydroxypicolinic acid	-4.36
Catechol	-3.99
Adenosine	-5.05
Isoleucine	-4.31
ZINC21882765	-7.59

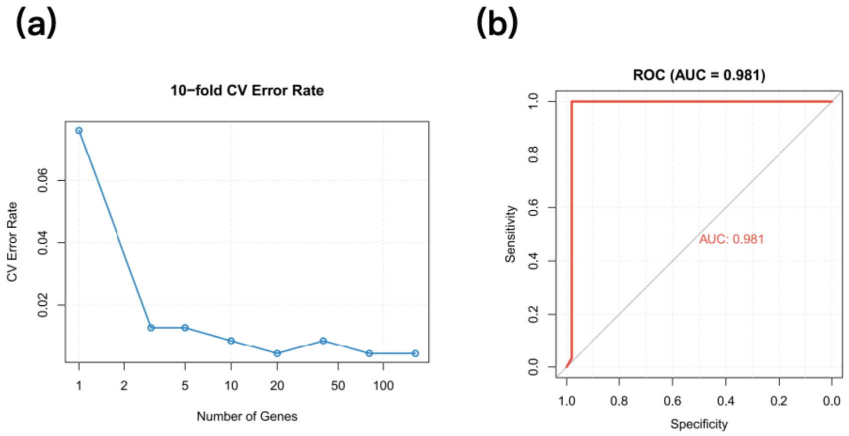

Figure S1. Rigorous validation of the random forest model (**a**) Cross-validation error as a function of the number of genes (**b**) ROC curves for the random forest model.
